# Mental Task Classification Scheme Utilizing Correlation Coefficient Extracted from Interchannel Intrinsic Mode Function

**DOI:** 10.1155/2017/3720589

**Published:** 2017-12-10

**Authors:** Md. Mostafizur Rahman, Shaikh Anowarul Fattah

**Affiliations:** Bangladesh University of Engineering and Technology (BUET), Dhaka 1000, Bangladesh

## Abstract

In view of recent increase of brain computer interface (BCI) based applications, the importance of efficient classification of various mental tasks has increased prodigiously nowadays. In order to obtain effective classification, efficient feature extraction scheme is necessary, for which, in the proposed method, the interchannel relationship among electroencephalogram (EEG) data is utilized. It is expected that the correlation obtained from different combination of channels will be different for different mental tasks, which can be exploited to extract distinctive feature. The empirical mode decomposition (EMD) technique is employed on a test EEG signal obtained from a channel, which provides a number of intrinsic mode functions (IMFs), and correlation coefficient is extracted from interchannel IMF data. Simultaneously, different statistical features are also obtained from each IMF. Finally, the feature matrix is formed utilizing interchannel correlation features and intrachannel statistical features of the selected IMFs of EEG signal. Different kernels of the support vector machine (SVM) classifier are used to carry out the classification task. An EEG dataset containing ten different combinations of five different mental tasks is utilized to demonstrate the classification performance and a very high level of accuracy is achieved by the proposed scheme compared to existing methods.

## 1. Introduction

Electroencephalogram (EEG) signal is used extensively nowadays by the researchers to handle different applications of brain-computer interface (BCI). EEG-based BCI systems employ electrical activity of brain to classify different EEG signals corresponding to various mental tasks precisely. One way to classify the signals effectively is to acquire discriminative features from that signal. As a matter of fact, different schemes to extract distinctive features are available in literature. For example, in [1], spectral power and asymmetry ratio based feature extraction scheme is proposed where an additional band (24–37 Hz) is used along with conventional lower spectral bands for mental task classification. This method offers comparatively satisfactory classification performance but lacks consistency for all cases. In [2], similar feature extraction scheme used in [1] is proposed; however, the difference is that it utilizes an additional high frequency band (40–100 Hz) to obtain those features. In [3], a dictionary consisting of power spectral density and common spatial pattern (CSP) algorithm is introduced to classify various mental tasks. Autoregressive (AR) model based feature extraction scheme is reported in [4] where sixth-order AR system is considered to extract feature. Moreover, in [5], multivariate AR models are taken into consideration and four different representations of AR coefficients are tested to classify mental task. In [6], feature extraction scheme based on sparse autoregressive model is investigated, which involves complex computation to exclude autoregressive coefficients that are useless in the prediction stage. In [7], a feature extraction method based on generalized Higuchi fractal dimension spectrum along with AR parameters is proposed. Wavelet transform and empirical mode decomposition (EMD) based classification methods are proposed in [8], where feature selection method is utilized for better classification performance. In [9], Stockwell transform based algorithm is proposed and mean square root of standard deviation of signal after transformation is utilized as distinctive feature. In [10], cross correlation based feature extraction scheme is introduced where cross correlation is computed between two channels keeping one of them always a fixed reference channel. Since one channel is kept fixed, the effect of considering cross correlation between all channels on overall feature quality has not been investigated. Moreover, unique choice of a fixed channel depends on various reasoning. Most of the reported algorithms except [10] are performed on the data taken from various channels while the interchannel relationship has not been utilized. It is considered that, for different types of task, different channels corresponding to different parts of the brain are stimulated. Measuring interchannel relationship in some efficient spectrotemporal domains may play a significant role to cover the spatial and temporal relationship between different channels. Thus, development of a proficient method capable of detecting and classifying different types of mental tasks utilizing the interchannel relationship is still undiscovered.

One of the main objectives of this paper is to extract robust feature by utilizing interchannel relationship. Instead of directly dealing with temporal EEG data, it is expected that features extracted from decomposed EEG data will provide more consistent characteristics. In particular, in this paper, widely used EMD is utilized to obtain intrinsic mode functions (IMFs) and first three IMFs are selected. Each IMF is utilized to compute the correlation coefficient from interchannel IMF data, which is referred to as inter-IMFCC method in this paper. Moreover, in the proposed method, unlike [10], only the channel information of the test frame is utilized to extract correlation coefficient and no previously defined reference signal is required for that purpose. Another objective of this paper is to observe whether the classification accuracy improves if different statistical features obtained from respective IMF are used along with inter-IMFCC. The support vector machine (SVM) classifier is used to carry out classification process. The effect of channel selection and that of using different kernels is investigated. Simulation details are reported considering a publicly available EEG dataset on various mental tasks.

## 2. Data Acquisition

A widely used EEG dataset collected by Keirn and Aunon is utilized [11] in this paper. EEG signals are acquired from the locations C3, C4, P3, P4, O1, and O2 which are denoted as the 10-20 international system of electrode placement. Measurements are made considering A1 and A2 as reference. Data are bandpass filtered using an analog filter with band limit of 0.1–100 Hz and sampled at 250 Hz with 12-bit quantizer. The recording is carried out for ten seconds during each session. EEG signals from seven subjects performing five different mental tasks, namely, geometrical figure rotation (R), mathematical multiplication (M), mental letter composing (L), visual counting (C), and baseline-resting (B) are investigated. For notational convenience, hereafter, each task is abbreviated with an alphabet as shown in the parentheses. However, data obtained from three subjects contain fewer than ten sessions or have some recording errors. Hence, like some other existing research works [1], in this paper, data from four subjects, each having ten or more sessions, are taken into consideration.

For the purpose of analysis of each ten-second session, a number of frames with shorter time interval are investigated as EEG signal is assumed to be nonstationary. In this case, one-second frame duration is considered with 0.5-second frame shift (i.e., 50% overlap between successive frames) [2], which provides reasonable number of samples (250 samples) in each frame.

## 3. Proposed Method

The proposed mental task classification scheme can be divided into four major steps: empirical mode decomposition, interchannel relation, feature extraction, and classification. These steps are described in detail in the following subsections.

### 3.1. Empirical Mode Decomposition

**Figure 1:**
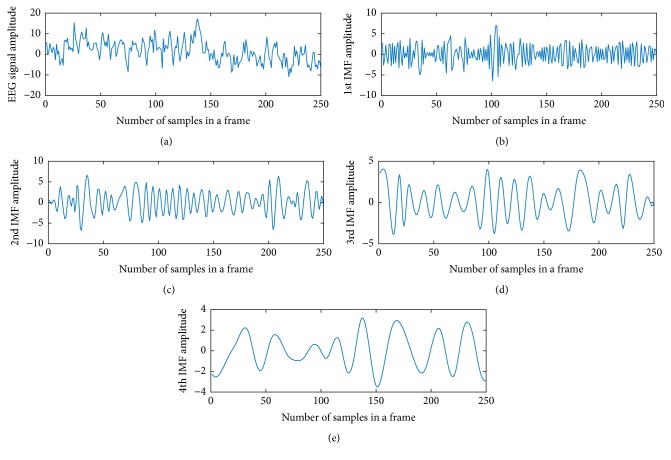
EEG signal and its IMFs.

Due to random nature of recordings of EEG data, it is very difficult to obtain discriminative characteristic from the time domain EEG data. Therefore, instead of directly utilizing EEG data, it may be easier to extract distinctive characteristic if decomposition is imposed on EEG data. Empirical mode decomposition is found very effective as it decomposes the signals in particular patterns preserving the originality of the signal. EMD is intuitive and adaptive, with IMFs directly derived from the signal under test without changing their domains. Moreover, each IMF contains information about how the frequency of the original signal changes in time. In Figure 1, a sample EEG signal and its four IMFs obtained from counting task are plotted. It is observed that the four IMFs, obtained after employing EMD on the test EEG signal, are lesser irregular and complex in nature than the original signal and have particular patterns. As a result, it is expected that classification performance will improve if IMFs are utilized to obtain distinctive features instead of main signal.

An IMF can be defined as a function which has equal number of maxima and minima or the difference between them is at most one. Moreover, the mean value of the envelope defined by the local maxima and the local minima is zero. In what follows, a brief description of obtaining IMFs by employing EMD on the EEG signal is described.

First, all the local maxima points of EEG data *y*_*i*_, obtained from channel *i*, are connected to define the upper envelope and all the local minima points are connected to define the lower envelope. The new signal *h*_1_[*n*] is reconstructed as

(1) $$\begin{array}{c} h_{1}\left[\;n\;\right]=y_{i}\left[\;n\;\right]-\mu_{1},\;n=1,2,3,\ldots ,N, \end{array}$$

where *μ*_1_ is the mean value of the envelopes and *N* is the number of samples of EEG signal, *y*_*i*_. The whole process is iterated *w* times until an IMF signal is generated according to the definition. The first IMF, *u*_1_, is defined by

(2) $$\begin{array}{c} u_{1}\left[\;n\;\right]=h_{w}\left[\;n\;\right],\;h_{w}\left[n\right]=h_{w-1}\left[n\right]-\mu_{w}, \end{array}$$

where *μ*_*w*_ is the mean value of the envelopes at *w*th iteration. The residue signal is found by subtracting the constructed IMF from the main signal; that is,

(3) $$\begin{array}{c} r_{1}\left[\;n\;\right]=y_{i}\left[\;n\;\right]-\;u_{1}\left[\;n\;\right]. \end{array}$$

This residue signal is considered as the main signal to estimate the next IMF. The process continues until the residue signal is either a signal consisting of a single maxima or minima or a constant value. Finally, *L* IMFs and a residue signal are generated after performing the whole decomposition process. For *L* level of decomposition, *y*_*i*_[*n*] can be reconstructed as

(4) $$\begin{array}{c} y_{i}\left[\;n\;\right]=\overset{L}{\underset{m=1}{\sum }}u_{m}\left[\;n\;\right]+r_{L}\left[\;n\;\right],\;n=1,2,3,\ldots ,N. \end{array}$$

Here *u*_1_, *u*_2_,…, *u*_*L*_ represent the IMFs.

In the proposed method, it is observed that the number of IMFs can be extracted considering any frame is four or more. It is expected that higher order IMFs which contain low frequency information may not be necessarily required while mental task is evaluated. Alternatively, it is expected to find more distinguishable characteristics in the IMFs that contain relatively high frequency information. As shown in Figure 1(e), it is clearly observed that fourth IMF contains very low frequency information. Considering all these facts, for the sake of consistency, each channel data of a given frame is decomposed into only three IMFs. However, effect of varying the number of IMFs on the classification performance is delineated next in the result section.

### 3.2. Interchannel Relation

In general, it is considered that, for different types of task, different spatial locations of brain, such as central, parietal, or occipital are stimulated. It is expected that data obtained from locations of the brain that are highly stimulated due to a specific type of task will be less correlated with data obtained from other less stimulated locations. For example, tasks involving visual effects are most likely to stimulate occipital regions predominantly. Therefore, EEG data obtained from the channels located in the occipital region will be significantly different from the data obtained from other less stimulated regions. Measuring interchannel relationship may play a significant role to cover this spatial and temporal relationship between different channels for a particular type of task. In the proposed method, correlation coefficient is utilized to measure interchannel relationship.

Correlation coefficient is a kind of statistical measure to quantify relationship between two or more signals. In this paper, it is utilized as a measuring tool to obtain interchannel correlation of *i*th and *j*th channel. Instead of directly using EEG data, correlation coefficient is obtained from the *m*th IMF, *u*_*m*_ decomposed from EEG signal. The correlation coefficient extracted from interchannel IMF data is referred to as inter-IMFCC in this paper. The inter-IMFCC *R*(*i*, *j*) obtained from *i*th and *j*th channel can be estimated as

(5) $$\begin{array}{c} R\left(\;i,j\;\right)=\frac{C\left(i,j\right)}{\sqrt{C\left(i,i\right)C\left(j,j\right)}}, \end{array}$$

where *C*(*i*, *j*) is the (*i*, *j*)th component of the covariance matrix **C** of the *i*th and *j*th channel IMFs *u*_*m*_^(*i*)^ and *u*_*m*_^(*j*)^; each consists of *N* samples. It is expressed as

(6) $$\begin{array}{c} C=\left[\begin{array}{cc} \mathrm{cov}\langle u_{m}^{\left(i\right)},u_{m}^{\left(i\right)}\rangle & \mathrm{cov}\langle u_{m}^{\left(i\right)},u_{m}^{\left(j\right)}\rangle \\ \mathrm{cov}\langle u_{m}^{\left(j\right)},u_{m}^{\left(i\right)}\rangle & \mathrm{cov}\langle u_{m}^{\left(j\right)},u_{m}^{\left(j\right)}\rangle \end{array}\right]. \end{array}$$

The covariance of *u*_*m*_^(*i*)^ and *u*_*m*_^(*j*)^ denoted by cov⁡〈*u*_*m*_^(*i*)^, *u*_*m*_^(*j*)^〉 is calculated considering the following formula:

(7) cov⁡umi,umj=1N−1∑n=1Numin−μi⋆umjn−μj.

Here *μ*_*i*_ and *μ*_*j*_ indicate the mean of IMF data obtained from *i*th and *j*th channels, respectively, and ⋆ denotes the complex conjugate. In the proposed method, all possible pair of *i*th and *j*th channels are taken into consideration to obtain inter-IMFCC which is expected to provide maximum utilization of channel information. However, effect of choosing lesser pairs of channels is also investigated and presented in the result section.

**Figure 2:**
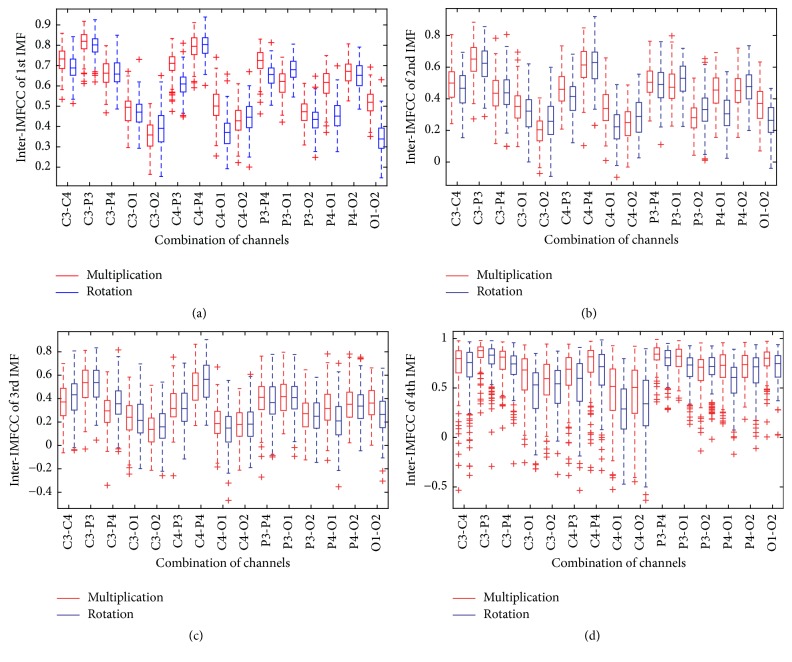
Inter-IMFCC obtained from different IMFs of subject 1.

One of the major advantages of utilizing inter-IMFCC as feature is that its values are bounded, which is |*R*(*i*, *j*)|<1. If the IMF data obtained from the channels are the same, inter-IMFCC is one; otherwise if there is no relationship, it is zero. To investigate the differentiating quality of inter-IMFCC as feature, a sample experiment considering multiplication and rotation task is performed. All fifteen different combinations of six channels denoted as C3-C4, C3-P3, C3-P4, and so forth are universally taken into consideration to measure inter-IMFCC. In Figure 2, the box plot corresponding to inter-IMFCC obtained for fifteen different combinations of channels is presented. The boxplot indicates various statistical information, such as median, 25th and 75th percentile, and outliers of inter-IMFCC. There are thirty boxplots in each subfigure; each boxplot represents inter-IMFCC measured from a particular combination of channel for a particular type of task performed by subject 1. In comparison to the boxplots presented in Figures 2(a), 2(b), and 2(c), the presence of outliers in boxplot presented in Figure 2(d) is much higher. As discussed before, higher order IMF contains very low frequency information which is less relevant to mental tasks considered here and hence poor distinctive features are expected to be extracted if 4th IMF is used. This fact is also reflected in boxplot presented in Figure 2(d). Therefore, in what follows, our discussions are restricted only for the first three IMFs.

It is observed that the values of inter-IMFCCs obtained for three combinations of channels, namely, C4-O1, P4-O1, and O1-O2, are found significantly higher in case of multiplication task than that in case of rotation task. It is to be noted that for these three combinations, O1 is considered as the reference channel obtained from left hemisphere and other nonreference channels, namely, C4, P4, and O2, are from right hemisphere. One possible reason behind this observation is that, in case of rotation task, O1 channel may get more stimulated than other channels due to the fact that, in rotation task, visually observed objects are required to rotate around their axis mentally. As a result, data obtained from O1 channel is less correlated with data obtained from other channels, specially the channels located in right hemisphere in case of rotation task. This observation corroborates the hypothesis that during performing a particular type of task if any location of the brain becomes more excited than other locations, data obtained from stimulated location will be significantly different from data obtained from comparatively less stimulated locations resulting in lower inter-IMFCC. Moreover, it is also found that inter-IMFCC values are comparatively higher in case of multiplication task than that obtained in case of rotation task. It is expected that, being an arithmetic task, multiplication involves more complexity in comparison to rotation task. As a result, all locations of brain get more excited while performing multiplication task than rotation task, which in turn leads to more correlation between channels in case of multiplication task. However, location of stimulation may vary from person to person depending on the nature of task.

### 3.3. Feature Extraction

In the proposed method, for the purpose of feature extraction, inter-IMFCCs are utilized to exploit the relationship among various channels. Moreover, statistical parameters such as root mean square (RMS), standard deviation, and entropy are also included in the feature vector to represent statistical measure of IMF data obtained from various channels. RMS depicts statistical measure of numerical values of varying quantity of the data obtained from channel *i* of corresponding IMF. For IMF data *u*_*m*_ consisting of *N* samples, RMS can be expressed as

(8) $$\begin{array}{c} \mathrm{RMS}\left(\;u_{m}^{\left(i\right)}\;\right)=\sqrt{\frac{1}{N}\overset{N}{\underset{n=1}{\sum }}u_{m}^{\left(i\right)}{\left[n\right]}^{2}},\;n=1,2,\ldots ,N. \end{array}$$

To measure the dispersion of the IMF data around its mean value *μ*_*i*_, standard deviation is proposed as a distinctive feature. Standard deviation of IMF data *u*_*m*_^(*i*)^ obtained from channel *i* is given by

(9) $$\begin{array}{c} \mathrm{std}\left(\;u_{m}^{\left(i\right)}\;\right)=\sqrt{\frac{1}{N}\overset{N}{\underset{n=1}{\sum }}{\left(u_{m}^{\left(i\right)}\left[n\right]-\mu_{i}\right)}^{2}},\;n=1,2,\ldots ,N. \end{array}$$

For the purpose of measuring uncertainty of the IMF data *u*_*m*_^(*i*)^, entropy is introduced in the feature vector. Entropy is a statistical measure of randomness that is defined as

(10) $$\begin{array}{c} \mathrm{ent}\left(\;u_{m}^{\left(i\right)}\;\right)=-\overset{N}{\underset{r=0}{\sum }}\left(\;p\langle \;u_{m}^{\left(i\right)}\left[\;r\;\right]\;\rangle \times \log_{2}\left(\;p\langle \;u_{m}^{\left(i\right)}\left[r\right]\;\rangle \;\right)\;\right), \end{array}$$

where *p*〈*u*_*m*_^(*i*)^[*r*]〉 indicates the probability of occurrence of a particular value *u*_*m*_^(*i*)^[*r*] of IMF data *u*_*m*_^(*i*)^ of *i*th channel and is denoted by

(11) $$\begin{array}{c} p\langle \;u_{m}^{\left(i\right)}\left[\;r\;\right]\;\rangle =\frac{n_{r}}{N}, \end{array}$$

and *n*_*r*_ indicates the number of occurrences of *u*_*m*_^(*i*)^[*r*] among the *N* number of samples of *u*_*m*_^(*i*)^; that is, ∑*n*_*r*_ = *N*.

In brief, for the purpose of feature extraction, at first, the raw EEG signal is preprocessed with a 60 Hz notch filter. After that, the eeg data corresponding to a channel is decomposed utilizing EMD where from each channel data three IMFs are extracted. Finally, the feature vector is formed utilizing inter-IMFCC and statistical parameters of IMFs, such as RMS, standard deviation, and entropy obtained from each channel. For *L* number of selected IMFs and *N*_*c*_ number of channels for each IMF, number of inter-IMFCCs obtained is *L* × ^*N*_*c*_^**C**_2_. The number of features obtained from statistical parameters of IMFs for a test frame is *L* × (*N*_*c*_ + *N*_*c*_ + *N*_*c*_). Finally the total feature dimension of the proposed method is *L* × (^*N*_*c*_^**C**_2_ + 3 × *N*_*c*_).

### 3.4. Classification

Classifier selection is essential to obtain satisfactory result while performing test validation of the proposed method. In the proposed method, kernel based SVM classifier is chosen to effectively classify mental tasks due to its effectiveness and acceptability in supervised classification. To generate an *N* dimensional decision vector $w={\left[\begin{array}{cccc} w_{1} & w_{2} & \cdots & w_{N} \end{array}\right]}^{T}$, features extracted from the IMF data are provided into the classifier instead of raw EEG data. The extracted features from the training dataset consisting of *P* frames are converted from the original space to a new representative vector space to discriminate different classes more efficiently. A class label is provided for each *N* dimensional *i*th frame **x**_*i*_ = *x*_*i*_[*n*], *n* = 1,…, *N*. For two class problems with two class labels +1 and −1, each frame **x**_*i*_ fulfills the following inequalities considering the threshold *b* [12]:

(12) wTxi+b≥+1,for  all  positive  xi,wTxi+b≤−1,for  all  negative  xi.

In kernel based SVM classifier, to match with class label of the training dataset, the following discriminant function *f*(**x**) is utilized to form the decision vector, which can be expressed as [12]

(13) $$\begin{array}{c} f\left(\;x\;\right)=\overset{P}{\underset{i=1}{\sum }}c_{i}K\left(\;x_{i},x\;\right)+b. \end{array}$$

Here *c*_*i*_ is an empirical value and kernel matrix **K** is given by

(14) $$\begin{array}{c} K=\left[\begin{array}{cccc} K\left(x_{1},x_{1}\right) & K\left(x_{1},x_{2}\right) & \cdots & K\left(x_{1},x_{P}\right)\\ K\left(x_{2},x_{1}\right) & K\left(x_{2},x_{2}\right) & \cdots & K\left(x_{2},x_{P}\right)\\ \vdots & \vdots & \cdots & \vdots \\ K\left(x_{P},x_{1}\right) & K\left(x_{P},x_{2}\right) & \cdots & K\left(x_{P},x_{P}\right) \end{array}\right]. \end{array}$$

For the purpose of classification, the performance of different kernel functions in SVM classifier is observed considering various feature extraction methods. It is found that polynomial kernel based classification outperforms other kernels in terms of classification accuracy. In all calculations of the reported classification accuracies, leave-one-out cross validation scheme is employed to generate classification result. In this scheme, each frame is tested one by one; that is, when a frame is left out for testing, remaining frames are used for training. Let us consider total *N*_A_ + *N*_B_ number of frames with *N*_A_ number of frames belonging to class A and *N*_B_ number of frames belonging to class B. In the leave-one-out cross validation scheme, when one of those *N*_A_ + *N*_B_ frames is left out for testing, remaining *N*_A_ + *N*_B_ − 1 frames are used for training. This process is repeated *N*_A_ + *N*_B_ times. Finally, classification accuracy is defined as the percentage of correctly identifying the class of each frame. Among total *N*_A_ + *N*_B_ number of frames if *N*_*t*_ number of frames are correctly classified, the classification accuracy can be expressed as

(15) $$\begin{array}{c} Accuracy=\frac{N_{t}}{N_{A}+N_{B}}\times 100\%. \end{array}$$

## 4. Simulation and Results

In this section, performance of various feature extraction methods is investigated considering classification accuracy obtained under different conditions, such as varying the feature dimension, utilizing different statistical parameters as feature, and use of various EEG channel locations. Moreover, effect of utilizing different kernel functions of SVM classifier on classification accuracy is also analyzed. A comparative analysis on classification performance between the proposed method and some other methods is also presented.

In the proposed method, instead of directly using channel data, corresponding IMFs are used to extract inter-IMFCC and statistical parameters using (4)–(10). Unless otherwise specified, polynomial kernel of SVM classifier is employed in leave-one-out cross validation manner to obtain classification accuracy. The classification task is carried out considering two types of mental tasks at a time, as conventionally done by other researchers [1, 2]. In this way, ten different combinations of the five types of tasks, as mentioned in Section 2, are possible. Here, for notational convenience, each combination of tasks is denoted with two alphabets from two different tasks. For example, MC refers to a two-class (multiplication and counting) classification problem, BL corresponds to another two-class (baseline-resting and mental letter composing tasks) classification problem. In what follows, detailed results and analyses are presented.

### 4.1. Effect of Variation of Number of IMFs

The number of IMFs to be used in the feature matrix directly dictates the feature dimension. It is already mentioned that higher order IMFs which contain very low frequency information are not necessary to be considered. The distinctive quality of the proposed inter-IMFCC feature deteriorates for 4th IMF as shown in Figure 2(d). Hence, in the proposed method, only first three IMFs are considered. In this subsection, effect of variation of number of IMFs is demonstrated on overall classification accuracy for four subjects. Here the number of IMFs is varied from 1 to 4 and different cases like extracting only one IMF (1IMF), two IMFs (2IMFs), and so forth are considered.

**Figure 3:**
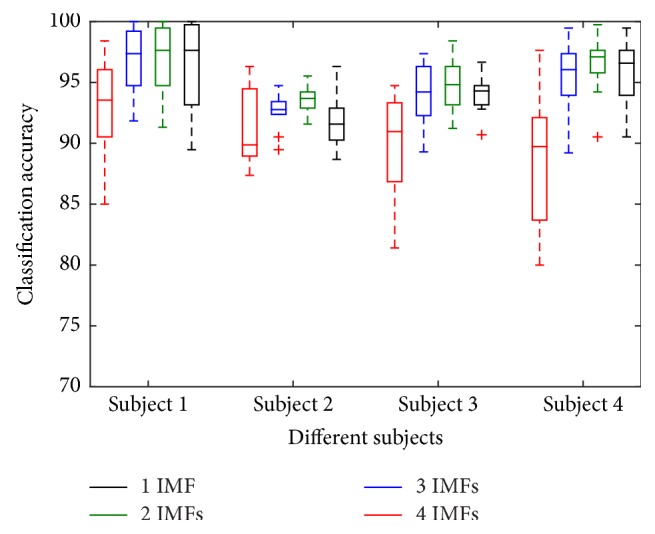
Effect of IMFs variation on classification accuracy for all four subjects.

In Figure 3, the box plot corresponding to classification performance obtained by varying number of IMFs is presented. The sixteen boxplots indicate various statistical information, such as median, 25th and 75th percentile, and outliers of classification accuracy. Each boxplot represents classification accuracy of ten different combinations of tasks for a subject considering particular number of IMFs to be used for feature extraction. It is found that, with the increase in number of IMFs, classification accuracy becomes more consistent for each subject until number of IMF is three. Moreover, it is observed that, for all subjects, features extracted considering the first three IMFs offer the best classification accuracy with respect to all other combinations of IMFs. That is why, although all channel data of a frame can be decomposed into four or more IMFs, only three IMFs are considered to extract feature. Meanwhile, considering three IMFs rather than four or higher number of IMFs offers a reduced feature dimension.

### 4.2. Effect of Different Statistical Feature

In the proposed method, as mentioned in Section 3.3, some statistical parameters are used as features, which are extracted from the channel IMF data. Effect of using conventional statistical features on classification accuracy is investigated considering ten widely used higher and lower order statistical parameters, namely, average (avg), median (med), mode (mod), maxima (max), minima (min), standard deviation (std), root mean square (RMS), entropy (ent), skewness (skew), and kurtosis (kurt). For notational convenience, hereafter, each statistical feature is abbreviated as shown in the parentheses. It is to be noted that the main objective of this paper is to demonstrate the efficacy of proposed correlation feature (inter-IMFCC) obtained from interchannel IMFs. It is expected that the use of proposed inter-IMFCC feature along with the conventional statistical features of IMFs will offer better classification performance. In this regard, two different cases are considered:

1. Use of only statistical features: each statistical feature is extracted from each of three IMFs of a channel, that is, for *N*_*C*_ number of channels with *L* number of IMFs extracted from each channel, feature dimension is *N*_*C*_ × *L*.
2. Use of proposed inter-IMFCC feature along with statistical feature: in this case, number of interchannel correlation coefficients (inter-IMFCC) to be obtained from *N*_*c*_ channels for each IMF is ^*N*_*c*_^**C**_2_. Hence, for *N*_*C*_ number of channels with *L* number of IMFs extracted from each channel, total feature dimension is (^*N*_*c*_^**C**_2_ + *N*_*C*_) × *L*.

**Figure 4:**
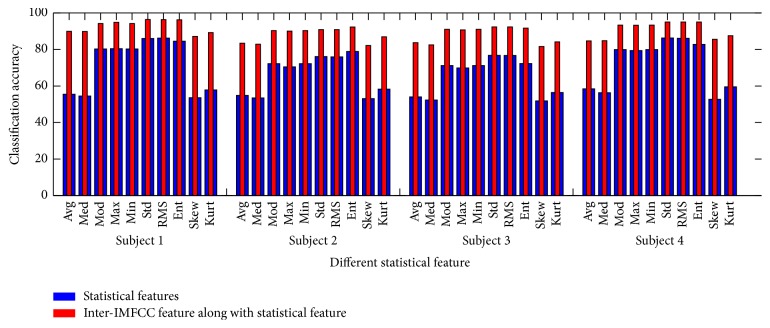
Effect of different statistical feature on classification accuracy for all four subjects.

In Figure 4, classification accuracies considering the previously discussed two cases for the ten statistical features obtained for all subjects are shown. It is observed that classification accuracy increases if inter-IMFCC is combined with channel statistical information of each IMF. Statistical parameters such as std, RMS, and ent of IMFs offer better classification performance compared to some higher order statistical feature, namely, skew and kurt. Moreover, features like max and min which are likely to be more biased because of the presence of noise are avoided. First order statistical parameters such as avg, med, and mod are also excluded as EEG signals are very random in nature. Due to distinctive nature of std, RMS, and ent, these three statistical parameters are finally chosen for the feature vector along with proposed inter-IMFCC feature to classify mental tasks.

### 4.3. Effect of Utilizing Kernel of SVM Classifier

**Figure 5:**
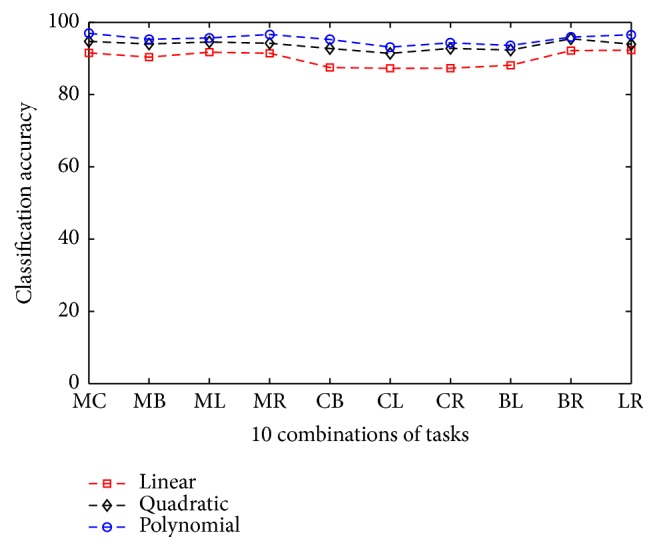
Classification accuracy obtained from four subjects considering different kernels in SVM classifier.

The effect of using different kernels in SVM classifier on overall classification performance of the proposed method is thoroughly investigated. In order to demonstrate the performance variation due to change in kernels, three widely used kernels are considered, namely, linear, quadratic, and polynomial kernel. To observe the variation of classification accuracies for different kernels, all 10 different combinations of tasks, namely, MC, MB, ML, MR, CB, CL, CR, BL, BR, and LR, from each subject are considered and average classification accuracy of those combination of tasks are measured from four subjects. In Figure 5, average classification accuracies for 10 different combinations of tasks by using three different kernels are plotted.

It is found that, between linear and quadratic kernel, the latter offers better classification performance. However, it is observed that the classification performances of polynomial kernel are consistently better in comparison to those obtained by linear and quadratic kernels in all cases. For that purpose, polynomial kernel of SVM classifier is chosen to classify the tasks in the proposed method.

### 4.4. Effect of Variation of Number of Channel Pairs

**Figure 6:**
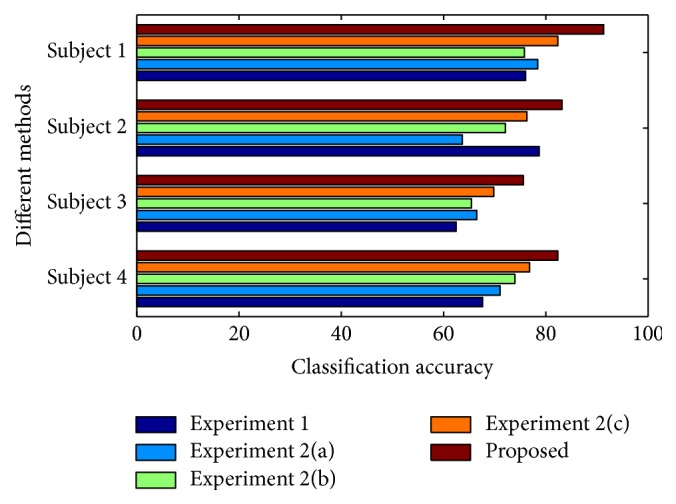
Effect of channel selection on classification accuracy for all four subjects in case of CB tasks.

In the proposed method, all possible pairs of channels are taken into consideration to obtain inter-IMFCC so that maximum channel information can be utilized. However, choosing lesser pairs of channels reduce feature size effectively. Reduction in feature size definitely helps in reducing computation time. Hence, effect of variation of the number of channel pairs is presented in this subsection. It is to be noted that, in [1, 2], asymmetry ratio of a pair of channels is computed considering one channel from left hemisphere and the other channel from right hemisphere. Similarly, in this paper, the effect of measuring inter-IMFCC considering one channel from left hemisphere and the other from right hemisphere is investigated. This investigation is performed considering counting and baseline-resting task and denoted as Experiment 1 in Figure 6. Moreover, the effect of measuring inter-IMFCC with respect to a specific region, denoted as Experiment 2, is also observed.

**Experiment 1 .**

For three channels located in left hemisphere C3, P3, and O1 and three channels located in right hemisphere C4, P4, and O2, possible nine combinations of computing inter-IMFCCs are (C3, C4), (C3, P4), (C3, O2), (P3, C4), (P3, P4), (P3, O2), (O1, C4), (O1, P4), and (O1, O2).

**Experiment 2 .**

Depending on the choice of region, such as parietal, central, or occipital, to obtain reference signals, three different investigations can be performed:

- Considering signals of parietal region as reference, eight combinations of channels for computing inter-IMFCCs are possible, such as (P3, C3), (P3, C4), (P3, O1), (P3, O2), (P4, C3), (P4, C4), (P4, O1), and (P4, O2).
- Considering signals of central region as reference, eight combinations of channels for computing inter-IMFCCs are possible, such as (C3, P3), (C3, P4), (C3, O1), (C3, O2), (C4, P3), (C4, P4), (C4, O1), and (C4, O2).
- Considering signals of occipital region as reference, eight combinations of channels for computing inter-IMFCCs are possible, such as (O1, C3), (O1, C4), (O1, P3), (O1, P4), (O2, C3), (O2, C4), (O2, P3), and (O2, P4).

In Figure 6, a comparative analysis among these experiments is presented in terms of classification accuracy. In the above two experiments, a reduced number of channel pairs are utilized and lower classification accuracy compared to the proposed method is achieved. As a result, it is not possible to select any one particular choice of reduced number of channels to obtain acceptable classification performance in all subjects.

### 4.5. Performance Comparison among Various Methods

With a view to comparing the classification performance, five methods referred to as PAR4, PAR5, PAR6, EF8, and EF3 have been considered. Among these five methods, three methods are based on power asymmetry ratio (namely, PAR*q*) computed from *q* number of spectral bands [1, 2]. Remaining two methods are based on EMD feature (namely, EF*n*) where *n* corresponds to number of features to be extracted from each IMF obtained by EMD decomposition [8].

In PAR*q* methods, depending on the number of frequency bands utilized, the methods are referred to as PAR4, PAR5, and PAR6. For example, in PAR4 method, features are extracted from the four traditionally used bands, namely, delta (<4 Hz), theta (4–7 Hz), alpha (8–13 Hz), and beta (14–20 Hz) while PAR5 utilizes an additional gamma band (23–37 Hz). In PAR6, one more additional band (40–100 Hz), along with these five bands, is proposed to compute power of spectral bands and asymmetry ratios. For one pair of channels, the asymmetry ratio for each spectral band is computed as [1]

(16) $$\begin{array}{c} A\left(\;i,j\;\right)=\frac{P\left(i\right)-P\left(j\right)}{P\left(i\right)+P\left(j\right)}, \end{array}$$

where two indices *i* and *j* are used to correspond to channel pairs placed in the left and right hemispheres, respectively. For example, *P*(*i*) corresponds to the spectral band power of the *i*th channel placed in the left hemisphere and *P*(*j*) corresponds to that obtained from the *j*th channel placed in the right hemisphere. Depending on the number of channels (*N*_*i*_ and *N*_*j*_) in each hemisphere, total *N*_*i*_ × *N*_*j*_ number of asymmetry ratios, denoted by *A*(*i*, *j*), can be computed for each spectral band. As a result, the feature dimension for PAR4, PAR5, and PAR6 method is *q* × *N*_*i*_ × *N*_*j*_ + *q* × (*N*_*i*_ + *N*_*j*_) where *q* denotes number of spectral bands considered for these methods.

On the other hand, in EF8 method, eight features are extracted from each IMF, namely, RMS, variance, Shannon entropy, Lempel-Ziv complexity measure, central frequency, maximum frequency, skewness, and kurtosis. However, in the proposed method, the first three of these eight statistical features are employed along with the proposed inter-IMFCC feature. In order to better demonstrate the effect of incorporating the inter-IMFCC feature, another method EF3 is considered where only the first three features are used without the proposed inter-IMFCC feature and classification performance of the EF3 method is also compared with that of the proposed method.

For the purpose of performance evaluation, leave-one-out cross validation technique is carried out in all methods. In Tables 1–4, the classification accuracies obtained by using four different subjects are separately reported for six methods. It is found that the classification accuracies obtained from different subjects are 90.5% or more in the proposed method. In all cases, it is observed that the proposed feature extraction method outperforms other existing methods reported in this paper in terms of classification accuracy. However, in some combinations of mental tasks, existing methods offer competitive classification performance with respect to proposed method. For example, in case of BR combination of subject 1 reported in Table 1, both EF8 and proposed method achieve 99.74% classification accuracy. In Table 4, it is observed that the average classification accuracies obtained by PAR6 and EF8 are very comparable with those obtained by the proposed method. For all subjects, it is found that the average classification accuracy obtained for EF8 is very close to EF3 despite having a larger feature dimension. However, after adding inter-IMFCC along with the three parameters used in EF3, the average classification accuracy increases drastically and for subject 2 and subject 3, it increases around 7.5% from EF3. In each reported existing method, it is observed that, for various combination of mental tasks, classification accuracy varies a lot. For example, in PAR4 method, for subject 1 and subject 4, the standard deviation of classification accuracies for various subjects is found to be 8.91% and 7.75% compared to 3.21% and 2.52% of the proposed method. It is found that the classification performance obtained by the proposed method varies from subject to subject, but not at a very large scale. For subject 2, the standard deviation obtained from different combination of mental tasks is found to be 1.16 which is the least among all four subjects. It is clearly observed that the proposed method offers consistently satisfactory classification accuracy in all cases irrespective of subjects and combination of mental tasks.

### 4.6. Computation Time

Average computational time is measured to extract features from one test signal for six methods, namely, PAR4, PAR5, PAR6, EF8, EF3, and proposed method. The whole process of computation is performed using Intel(R) Core(TM) i5-4200M processor with 2.50 GHz clock speed and 4 GB ram. The feature dimension and the feature extraction time for six methods are listed in Table 5.

It is found that the proposed method uses a very small computation time for feature extraction compared to recently reported EF8 method. One of the reasons for such a small computation time for the proposed method is its feature dimension compared to EF8. For three selected IMFs and six channels for each IMF, the feature dimension of the proposed method is 3 × (^6^**C**_2_ + 3 × 6) = 99. On the contrary, for four selected IMFs and similar number of channels for each IMF, the feature dimension of the EF8 and EF3 method is 4 × (8 × 6) = 192 and 4 × (3 × 6) = 72, respectively. In case of PAR4, PAR5, and PAR6, feature dimension is 4 × 3 × 3 + 4 × 6 = 60, 5 × 3 × 3 + 5 × 6 = 75, and 6 × 3 × 3 + 6 × 6 = 90, respectively. The PAR4, PAR5, and PAR6 method utilizes lesser time and features but the classification accuracies are lesser in these methods than proposed method.

## 5. Conclusion

In the proposed mental task classification scheme, interchannel correlation coefficient of each IMF is utilized to explore the relationship between channels, which is referred to as inter-IMFCC method. Moreover, intrachannel features, such as standard deviation, RMS, and entropy of each IMF, are also measured. Finally, both interchannel features and intrachannel features of each IMF are utilized to form feature vector and a quite satisfactory classification performance is achieved. It is observed that increase in feature dimension by considering more IMFs not necessarily provides better classification performance and thus only three IMFs from each channel are found sufficient. Effect of selecting different combinations of channels is also investigated and it is observed that considering all combinations of channels provides the best classification performance irrespective of the tasks or the subjects. Classification performance for various feature extraction methods is listed considering polynomial kernel and it is observed that the proposed method outperforms other methods in terms of classification accuracy. Results obtained from various types of investigation verify that the proposed mental task classification scheme is capable of classifying EEG signals with high classification accuracy.

## Figures and Tables

**Table 1:** Overall classification accuracy obtained for subject 1.

Task	PAR4 [1]	PAR5 [1]	PAR6 [2]	EF8 [8]	EF3	Proposed
MC	88.42	88.95	91.58	96.32	96.32	97.89
MB	82.11	83.95	87.37	95.79	90.53	97.37
ML	86.05	87.37	90.26	98.68	96.84	99.47
MR	89.47	91.05	93.95	98.95	97.63	100.00
CB	72.37	78.95	82.63	92.11	92.11	98.95
CL	65.26	69.74	77.11	84.47	83.95	91.32
CR	71.58	74.74	80.26	86.32	81.58	91.84
BL	69.74	71.05	82.89	90.53	86.58	94.74
BR	83.42	86.58	92.37	99.74	97.89	99.74
LR	70.26	77.37	81.84	94.47	89.21	95.53

Avg	77.87	80.97	86.03	93.74	91.26	96.68
Std dev	8.91	7.65	5.82	5.30	5.92	3.21

**Table 2:** Overall classification accuracy obtained for subject 2.

Task	PAR4 [1]	PAR5 [1]	PAR6 [2]	EF8 [8]	EF3	Proposed
MC	69.47	76.58	83.42	82.37	80.79	93.95
MB	78.95	86.32	91.05	83.16	84.74	93.16
ML	70.53	83.42	87.63	90.79	88.42	95.00
MR	71.32	79.21	90.00	85.79	82.63	92.89
CB	74.74	80.53	92.11	82.89	84.74	92.63
CL	64.74	75.53	89.47	91.32	92.37	93.95
CR	68.68	73.42	87.11	79.74	84.47	93.42
BL	71.84	79.47	86.84	87.89	89.47	94.21
BR	76.05	79.74	86.05	85.53	84.47	91.58
LR	71.58	80.79	84.21	88.16	85.79	95.53

Avg	71.79	79.50	87.79	85.76	85.79	93.63
Std dev	4.01	3.74	2.85	3.79	3.41	1.16

**Table 3:** Overall classification accuracy obtained for subject 3.

Task	PAR4 [1]	PAR5 [1]	PAR6 [2]	EF8 [8]	EF3	Proposed
MC	68.42	74.91	79.82	86.32	88.25	96.32
MB	73.16	74.56	81.40	85.79	86.49	93.68
ML	71.58	74.39	80.70	85.96	86.32	92.46
MR	74.74	79.47	87.89	92.63	87.89	96.49
CB	70.53	72.98	81.05	87.89	85.26	91.23
CL	72.81	77.19	80.35	88.60	83.68	93.16
CR	68.60	75.44	81.40	92.63	90.00	94.91
BL	74.39	74.56	84.21	87.54	83.68	94.91
BR	73.86	75.96	84.21	92.28	85.61	94.74
LR	77.89	83.33	87.54	92.98	93.33	98.42

Avg	72.60	76.28	82.86	89.26	87.05	94.63
Std dev	2.92	3.05	2.96	3.03	2.96	2.11

**Table 4:** Overall classification accuracy obtained for subject 4.

Task	PAR4 [1]	PAR5 [1]	PAR6 [2]	EF8 [8]	EF3	Proposed
MC	83.95	90.53	97.63	99.47	98.42	99.74
MB	86.84	90.53	94.74	96.84	95.00	97.11
ML	86.05	88.42	92.89	95.00	92.89	95.79
MR	84.21	88.95	93.95	92.63	91.32	97.11
CB	81.58	82.37	86.58	94.21	95.26	98.16
CL	78.16	81.58	87.63	85.79	87.89	94.21
CR	88.68	92.89	96.32	95.26	92.63	97.11
BL	68.16	77.63	83.95	85.26	86.58	90.53
BR	94.47	95.26	97.11	97.89	93.95	97.63
LR	94.47	96.05	97.37	96.05	93.16	96.84

Avg	84.66	88.42	92.82	93.84	92.71	96.42
Std dev	7.75	6.09	4.99	4.78	3.48	2.52

**Table 5:** Feature dimension and average time for feature extraction.

Different methods	PAR4 [1]	PAR5 [1]	PAR6 [2]	EF8 [8]	EF3	Proposed
Feature dimension	60	75	90	192	72	99
Average time (ms)	52.63	60.02	76.36	2749.30	128.80	108.11
